# Development of SSR markers for *Astragalus lehmannianus*, a vulnerable species from northwestern China

**DOI:** 10.1002/aps3.11297

**Published:** 2019-10-24

**Authors:** Mingying Wu, Xiaojun Shi, Dunyan Tan

**Affiliations:** ^1^ Xinjiang Key Laboratory of Grassland Resources and Ecology and Ministry of Education Key Laboratory for Western Arid Region Grassland Resources and Ecology College of Grassland and Environment Sciences Xinjiang Agricultural University Ürümqi 830052 People's Republic of China; ^2^ College of Biology and Environmental Sciences Jishou University Jishou 416000 People's Republic of China

**Keywords:** Astragalus lehmannianus, conservation, Fabacaeae, microsatellite marker, vulnerable species

## Abstract

**Premise:**

*Astragalus lehmannianus* (Fabaceae) is a vulnerable species found in the cold deserts of northwestern China. We aimed to characterize polymorphic microsatellite loci for *A. lehmannianus* to support future studies of population genetic dynamics and conservation management of the species.

**Methods and Results:**

We used next‐generation sequencing to detect polymorphic microsatellites. Twenty‐five potential microsatellite loci were identified, 12 of which were polymorphic and present in the three study populations of *A. lehmannianus*. Levels of observed and expected heterozygosities were 0.000–1.000 and 0.000–0.827, respectively. Furthermore, two and five of the 12 developed primers were successfully amplified in two congeneric species, *A. arpilobus* and *A. oxyglottis*, respectively.

**Conclusions:**

These newly developed microsatellite markers can be used to determine population diversity and to develop conservation strategies in *A. lehmannianus*.

*Astragalus lehmannianus* Bunge (Fabaceae) is a vulnerable perennial herb in China that is distributed only on stationary and semi‐stationary dunes on the southern margin of the Gurbantunggut Desert in Xinjiang Uygur Autonomous Region (XJ), northwestern China (Fu, 1993; Shen, 2011). This species is threatened by anthropological disturbances and has been listed in the first group of key protected plants in XJ (Li, 2015). An extensive field survey covering the distribution area of *A. lehmannianus* in China was made from 2013 to 2017, and only six populations were confirmed to be extant. Furthermore, the locations of *A. lehmannianus* populations are fragmented and isolated from each other. Although the biochemistry and seed predation of this species have been reported (Mestechkina et al., 2000; Han et al., 2018), genetic information remains unknown.

Fragmented endangered species with restricted geographic distribution tend to have low levels of genetic diversity within populations, and restricted gene flow and increased genetic differentiation among populations (Wall et al., 2014). If endangered and vulnerable species are unprotected, their lower genetic diversity can significantly decrease the chances of survival in changing environments (Frankham, 2010). Effective protection of rare and endangered species often takes advantage of the genetic diversity information of the species (Selyutina et al., 2016).

Microsatellite markers developed in *A. holmgreniorum* Barneby (King et al., 2012), *A. camptodontus* Franch. (Zhang et al., 2013), *A. mongholicus* Bunge (Wang et al., 2013), *A. michauxii* (Kuntze) F. J. Herm. (Wall et al., 2014), and *A. bibullatus* Barneby & E. L. Bridges (Morris et al., 2016) were not found to be polymorphic for *A. lehmannianus* in preliminary tests. Therefore, we developed 12 variable microsatellite markers using the restriction site–associated DNA sequencing (RAD‐Seq) approach from the genome of *A. lehmannianus* and also tested the transferability of these markers to the congeneric species *A. arpilobus* Kar. & Kir. and *A. oxyglottis* Steven ex M. Bieb. These markers will provide useful tools for population genetics and demographic history of current populations of *A. lehmannianus* and related species. Furthermore, the study of genetic diversity using these markers will contribute to the formulation of conservation strategies for *A. lehmannianus*.

## METHODS AND RESULTS

Total genomic DNA was extracted from dried leaves of *A. lehmannianus* plants collected from three natural populations in XJ (Jimsar County [G], Fukang City [C], Jimsar County [J]; Appendix 1) on the southern margin of the Gurbantunggut Desert, following the cetyltrimethylammonium bromide (CTAB) method of Doyle and Doyle (1987) with the centrifugal time extended for 3–5 min.

The RAD‐Seq libraries were created by Huashibaiao Biotech Company (Beijing, China) to generate the simple sequence repeat (SSR) information. Our selections were di‐, tri‐, and tetranucleotide motifs with flanking regions larger than 100 bp and a minimum of 10, six, and four repeats, respectively. A total of 165 primer pairs, which were designed using Primer Premier 5.0 (PREMIER Biosoft International, Palo Alto, California, USA) and synthesized by Sangon Biotech (Shanghai, China), were obtained and tested for amplification. We attached the universal M13 sequence (TGTAAAACGACGGCCAGT) tag to the 5′ end of the forward primer using the method described by Schuelke (2000) to label the products with fluorescence. Designed primers were tested on 65 samples of *A. lehmannianus*. To examine cross‐species amplification, 10 individuals each of *A. arpilobus* and *A. oxyglottis* were collected from the Gurbantunggut Desert in the same general locality from which samples of *A. lehmannianus* were collected. PCR was performed in a total volume of 25 μL containing 1 μL of template DNA with an initial concentration of 100 ng/μL, 7.9 μL of ddH_2_O, 12.5 μL of 2× Power Taq PCR MasterMix (TIANGEN, Beijing, China), 0.4 μL of TP‐M13 forward primer, and 1.6 μL of each reverse primer and universal M13 sequencing primer labeled with either HEX or 6‐FAM. All candidate primer pairs were tested by a touchdown PCR protocol in the following procedure: 94°C for 4 min; 10 cycles of 94°C for 40 s, 65–55°C (Δ1°C touchdown per cycle) for 50 s, 72°C for 40 s; 30 cycles of 94°C for 40 s, 55°C for 45 s, 72°C for 1 min; and a final extension at 72°C for 6 min. The amplified products were tested on 1.5% agarose gel and 6% polyacrylamide gel. The size of the amplified fragments was measured with an ABI 3730xL Genetic Analyzer (Applied Biosystems, Waltham, Massachusetts, USA), and allele sizes were scored using the software GeneMarker version 1.51 (Applied Biosystems).

Twelve polymorphic microsatellite markers were developed and used on 65 individuals of *A. lehmannianus* from different populations (Table 1). An additional 13 monomorphic microsatellite markers were developed but not used (Appendix 2). Population genetic parameters, including number of alleles, levels of observed and expected heterozygosity, the inbreeding coefficient, and departure from Hardy–Weinberg equilibrium, were calculated using GenAlEx version 6.5 software (Peakall and Smouse, 2012). Linkage disequilibrium was analyzed with GENEPOP version 4.6.9 (Rousset, 2008).

**Table 1:** Characteristics of the 12 polymorphic microsatellite loci developed in *Astragalus lehmannianus*

Locus^a^	Primer sequences (5′–3′)	Repeat motif	Allele size range (bp)	Fluorescent dye	GenBank accession no.
AL23	F: AGTGCCTGAACAGTTACAAACCAAGA	(TC)_20_	146–162	6‐FAM	MK606235
	R: GGAAGAAAATGCAGCCATCA				
AL26	F: CTATAATAGCGGTGATGAAGGGAAC	(GA)_25_	132–158	6‐FAM	MK606236
	R: GTACCCGCGAGAAATCTTGTT				
AL35	F: GGTTTTAAACGTGAATTTAGTTGCC	(TA)_12_	143–159	6‐FAM	MK606237
	R: GTCTTTCTTTTAAAGATGTTTTC				
AL41	F: CCATAGAGATTTTAGGTTCAG	(TA)_20_	128–156	HEX	MK606238
	R: CAGTATGTTTTGGTGTATT				
AL42	F: AACAAGCAATCTATGTGCCCC	(AAT)_10_	139–154	6‐FAM	MK606239
	R: TCGAACCTAACTCAGACTGCA				
AL43	F: CAAGCAACTGTATTGGTGCA	(TA)_13_	144–154	HEX	MK606240
	R: CCTTCCACTACACATTTCAC				
AL50	F: CCCATTACAAGATATATTGCC	(AT)_16_	148–164	6‐FAM	MK606241
	R: GCAAGAATCCTGCCGATTTT				
AL52	F: TTCAGGAGCACTGCAAAGGTT	(TA)_17_	137–167	6‐FAM	MK606242
	R: GAGTTAGCATCTAAGAAAACA				
AL53	F: AATTCCTTTCAGATGGGAGC	(AG)_12_	130–150	6‐FAM	MK606243
	R: TTGCTTAAAGCTTGCTGCCT				
AL54	F: TCAGAGGGTCTAATTGGCTTC	(TA)_17_	130–150	HEX	MK606244
	R: AGTTAGGCGTTAGCCTTACGT				
AL59	F: AAAAGGATTGACCAATTCAGC	(CA)_15_	122–156	6‐FAM	MK606245
	R: GGAAAGAAGGCAATTATGCTC				
AL65	F: AAGCGACATTTTGTCCTGTC	(TA)_18_	140–148	6‐FAM	MK606246
	R: CGTAAAAAATTAAAAGCGG				

a A touchdown PCR protocol was used rather than locus‐specific annealing temperatures.

The 12 loci were polymorphic in the three populations, with two to 11 alleles (mean 5.17) in population G, two to 10 alleles (mean 4.92) in population C, and one to five alleles (mean 2.83) in population J. The levels of mean observed and expected heterozygosities, respectively, were 0.507 (range 0.107–0.893) and 0.566 (range 0.249–0.827) in population G, 0.547 (range 0.094–0.938) and 0.505 (range 0.253–0.792) in population C, and 0.567 (range 0.000–1.000) and 0.497 (range 0.000–0.800) in population J (Table 2). A total of nine loci in population G and eight loci in population C deviated from Hardy–Weinberg equilibrium. This deviation may be due to inbreeding or a low number of samples selected for the study. There was no evidence of linkage disequilibrium between any loci in any of the three populations, indicating that all of the loci were independent and could potentially be used in studies of genetic diversity.

**Table 2:** Measures of genetic variation for the 12 polymorphic microsatellite loci in three populations of *Astragalus lehmannianus*.^a^

Loci	Population G (*n* = 28)				Population C (*n* = 32)				Population J (*n* = 5)			
	*A*	*H*_o_	*H*_e_ b	Amplification success (%)	*A*	*H*_o_	*H*_e_ b	Amplification success (%)	*A*	*H*_o_	*H*_e_ b	Amplification success (%)
AL23	7	0.357	0.783***	100	5	0.381	0.371^ns^	100	2	0.800	0.480^ns^	100
AL26	4	0.250	0.336***	100	4	0.188	0.253***	100	2	0.800	0.480^ns^	100
AL35	3	0.143	0.249***	100	4	0.375	0.322^ns^	100	1	0.000	0.000	60
AL41	6	0.893	0.726**	100	7	0.938	0.678***	100	5	1.000	0.800^ns^	100
AL42	2	0.464	0.448^ns^	100	2	0.438	0.430^ns^	100	2	0.400	0.320^ns^	100
AL43	3	0.107	0.331***	100	3	0.094	0.274***	100	3	0.400	0.340^ns^	100
AL50	6	0.444	0.604***	96.8	7	0.594	0.628^ns^	100	3	0.400	0.460^ns^	100
AL52	9	0.393	0.814***	100	7	0.125	0.604***	100	3	0.000	0.640*	100
AL53	2	0.893	0.499***	100	2	0.906	0.500***	100	3	0.800	0.580^ns^	100
AL54	7	0.821	0.827^ns^	100	6	0.844	0.715***	100	3	0.400	0.620^ns^	100
AL59	11	0.786	0.788^ns^	100	10	0.844	0.792*	100	5	1.000	0.760^ns^	100
AL65	2	0.536	0.392***	100	2	0.938	0.498***	100	2	0.800	0.480^ns^	100
Mean	5.17	0.507	0.566	99.7	4.92	0.547	0.505	100	2.83	0.567	0.497	96.7

Note

*A* = observed number of alleles; *H*_e_ = expected heterozygosity; *H*_o_ = observed heterozygosity; *n* = number of individuals sampled.

a Voucher and locality information are provided in Appendix 1.

b Deviation from Hardy–Weinberg equilibrium: *P < 0.05, **P < 0.01, ***P < 0.001, ns = not significant.

Cross‐species amplification using the microsatellites developed for *A. lehmannianus* was successful for two of the 12 loci (AL42 and AL53) in *A. arpilobus* and for five of the 12 loci (AL26, AL41, AL50, AL52, and AL54) in *A. oxyglottis* (Table 3). This weakly successful cross‐species amplification may be due to the relatively high genetic differentiation among the three species.

**Table 3:** Results of cross‐amplification (allele size ranges) for the 12 polymorphic microsatellite markers developed for *Astragalus lehmannianus* in two related *Astragalus* species.^a^

Locus	*A. arpilobus*	*A. oxyglottis*
AL23	—	—
AL26	—	134–136
AL35	—	—
AL41	—	151–157
AL42	138–144	—
AL43	—	—
AL50	—	134
AL52	—	136
AL53	136	—
AL54	—	130–148
AL59	—	—
AL65	—	—

Note

— = failed amplification.

a Locality and voucher information are provided in Appendix 1.

## CONCLUSIONS

Our results provide 12 microsatellite markers for *A. lehmannianus* that can serve as a useful resource for estimation of genetic diversity. These newly developed primers can be used for conservation and restoration strategies of this rare species.

## AUTHOR CONTRIBUTIONS

M.Y.W., D.Y.T., and X.J.S. conceived and designed the experiments. M.Y.W. performed the experiments. M.Y.W. and X.J.S. analyzed the data. M.Y.W., D.Y.T., and X.J.S. contributed to manuscript preparation. All authors reviewed and approved the manuscript.

## Data Availability

All sequence information was uploaded to the National Center for Biotechnology Information (NCBI) Sequence Read Archive (accession no. PRJNA547861); primer sequences were uploaded to GenBank (accession no. MK606235–MK606246; Table 1).

## APPENDIX 1 Locality and voucher information for the sampled *Astragalus* populations in this study.

**Table:**

Species^a^	Collection locality^b^	Locality code	Geographic coordinates	*n*	Voucher no.^c^
*Astragalus lehmannianus* ^1,2^ Bunge	Jimsar County	G	44°23′53″N, 88°47′13″E	28	XM201720
	Fukang City	C	44°22′19″N, 88°08′28″E	32	XM201739
	Jimsar County	J	44°25′27″N, 88°56′43″E	5	XM201686
*A. arpilobus* ^3^ Kar. & Kir.	Fukang City	FK	44°22′45″N, 88°10′02″E	10	QJ201811
*A. oxyglottis* ^3^ Steven ex M. Bieb.	Jimsar County	JM	44°23′25″N, 88°47′33″E	10	MY201849

Note

*n* = number of individuals sampled.

a Samples were used as follows: 1 = individuals used for pre‐screening analyses and genotyping tests; 2 = individuals used for characterization of microsatellites; 3 = individuals used for cross‐amplification tests.

b All samples were collected from Xinjiang Uygur Autonomous Region in China.

c Vouchers deposited at Xinjiang Agricultural University (XJA), Xinjiang, Xinjiang Uygur Autonomous Region, China.

## APPENDIX 2 Characteristics of 13 monomorphic microsatellite loci developed and tested in *Astragalus lehmannianus*.

**Table:**

Locus	Primer sequences (5′–3′)	Repeat motif	Allele size (bp)	GenBank accession no.
NS‐AL‐08	F: GACGTATTTGTCCCCCAAAA	(AG)_15_	280	MN159044
	R: CACTTTTTCTTTTCCTCTTTCTCAA			
NS‐AL‐47	F: GTCAAAAGCATCGAGTTTCCA	(AG)_10_	115	MN159045
	R: ATTCGGATGAGATTTCCGTG			
NS‐AL‐49	F: TTCCAACATGCTCATAACTTC	(GA)_10_	116	MN159046
	R: TCAACAACGCCATGGACA			
NS‐AL‐62	F: CCTTTAACGTTGGAAACAA	(GAT)_18_	149	MN159047
	R: ACAGATGCTGAAGACGTCTCA			
NS‐AL‐70	F: ATGAAGGGCGTGTTTGTTTC	(TC)_10_	272	MN159048
	R: CGGGGTCATAGGACTCTCTG			
NS‐AL‐85	F: GGAATTCCTCGTGCATCTGT	(GT)_10_	270	MN159049
	R: CAAATTCCCATTCCATCACA			
NS‐AL‐97	F: ATTTTTATCCCGCACCCTTC	(CT)_10_	268	MN159050
	R: TGTCATCTTCACCCCATGAA			
NS‐AL‐98	F: CATTTCCTCTCTCTCCGGC	(CT)_10_	268	MN159051
	R: AGCCCAGTTCGTTTTCTGTG			
NS‐AL‐121	F: TCTCATCACTCTCTGCCCCT	(GA)_10_	278	MN159052
	R: TGGATTTCATATTCTACAACCTTTTG			
NS‐AL‐129	F: GTACCGAATCCAACTCCGAA	(GA)_13_	277	MN159053
	R: TCGTTCTGCTTCCTTCGTTT			
NS‐AL‐137	F: TGATCTTCCAGCATGTTCTCA	(GA)_11_	276	MN159054
	R: CGTTTGCGAAAACAGATACG			
NS‐AL‐142	F: GTTTCCATGTGTGTGTGCG	(AG)_15_	275	MN159055
	R: CCCAAAATAACGAAGCCAAA			
NS‐AL‐143	F: ACTGGAATTCCCCTTCCAAC	(GT)_10_	275	MN159056
	R: TGAGGAAGAAGAGGGTCGTG			
